# Displacement of the Recurrent Laryngeal Nerve in Patients with Recurrent Goiter Undergoing Redo Thyroid Surgery

**DOI:** 10.1155/2018/4763712

**Published:** 2018-02-28

**Authors:** Emin Gurleyik, Fuat Cetin, Sami Dogan, Erman Yekenkurul, Ufuk Onsal, Fatih Gursoy, Alper Ipor

**Affiliations:** Department of Surgery, Medical Faculty, Düzce University, Düzce, Turkey

## Abstract

Thyroid reoperations are surgically challenging because of scarring and disturbances in the anatomy of the recurrent laryngeal nerve (RLN). This study was conducted on 49 patients who underwent redo surgery. 61 RLNs were identified and completely exposed. Their functional integrity was evaluated using intraoperative nerve monitoring (IONM). Indications for secondary surgery, anatomical changes secondary to recurrent goiter mass and prior surgery, and results of IONM were studied. Frequent indications for redo surgery were multinodular goiter (MNG) in 19 (38.8%) and results of cytology in 14 (28.5%) patients. The mean time interval between primary and redo thyroid surgery was 23.4 years. We laterally approached 41 (67.2%) thyroid lobes between the sternocleidomastoid and sternohyoid muscles. 16 (26.2%) RLNs were found to be adherent to the lateral surface of the corresponding thyroid lobe. The functional integrity of all RLNs was confirmed by IONM. The remnant thyroid tissue can then lead to goiter recurrence requiring secondary surgery after a long period of time. The indications for redo surgery were similar to primary cases. Lateral displacement of the RLN which is adherent to the lateral surface of recurrent goiter mass is common anatomic variation. Thyroid reoperations based on awareness of anatomical disturbances can be performed safely by an experienced surgeon with support of ancillary electrophysiological technology.

## 1. Introduction

The resection of an enlarged remnant gland and reoperations in the thyroid bed are surgically challenging because of the distortion of anatomic planes and scarring from prior surgery [1]. The recurrent laryngeal nerve (RLN) is the most important structure for postoperative complications of thyroid surgery. Visual identification of the RLN is mandatory during thyroid surgery for preventing nerve injury and preserving nerve integrity. However, RLN visualization is not easy while dissecting scar tissue. Therefore, the risk of RLN injury is the major concern of reoperative thyroid surgery. Such procedures are technically difficult and involve a higher risk of complications than do primary procedures [2, 3]. Thyroid reoperations can be challenging, even for a highly experienced thyroid surgeon, as visual identification of the RLN is more difficult during dissection of scar tissue than in the virgin neck [4]. Disturbances in the anatomy and changes in the cervical course of the nerve due to both recurrent goiter mass and wound contraction during the scar formation process after primary surgery are other challenging factors in redo surgery. Intraoperative nerve monitoring (IONM) is a widely accepted adjunctive method for visual identification of the RLN during surgical dissection. In redo surgery, IONM seems to be more useful for making the functional identification of the nerve, as well as for checking its integrity.

We studied the effect of prior surgery and recurrent goiter on the surgical anatomy of the thyroid gland and especially on the cervical course of the RLN in patients with redo thyroid surgery.

## 2. Patients and Methods

Hospital records of thyroidectomy cases in the last five years were retrospectively reviewed to determine reoperations for the treatment of recurrent goiter. A total of 52 patients with thyroid reoperations were retrieved after chart review. We aimed to study the effects of recurrent goiter mass and previous surgery on the surgical anatomy and the cervical course of the RLN. Preoperative laryngoscopy showed unilateral vocal cord (VC) palsy after primary surgery in three patients who were excluded from the study. This study was conducted with the remaining 49 patients who had recurrent goiter and underwent secondary surgery. 61 RLNs (38 right and 23 left) were identified, and their cervical courses were exposed until the point of laryngeal entry. The functional identification and integrity of these nerves were evaluated and confirmed using the IONM. 

*RLN Dissection Technique.* Dissection was performed using a binocular loupe (magnification, ×2.5). After freeing and medially mobilizing lobes of the thyroid gland, the RLN was searched in anterior and posterior adjacent planes to the thyroid. The nerve was identified and fully isolated under the guide of electrophysiological monitoring on both sides using a conventional lateral approach. 

*Intraoperative Neuromonitoring of the RLN.* We performed IONM to functionally identify the nerve and to determine motor function and functional integrity of the RLN. Nerve monitoring was performed using the Nerve Integrity Monitor (NIM Response 3.0 System; Medtronic Xomed, Jacksonville, FL, USA). The setup of the device was 1 mA as stimulation intensity and 100 *μ*V as amplitude threshold. Standard IONM was performed as a four-step procedure:

V1: vagus nerve (VN) stimulation before identification of the RLN (VN predissection)
R1: RLN stimulation when first identified in the dissection field (RLN predissection)
R2: RLN stimulation after complete dissection of the lateral thyroid lobe (RLN postdissection)
V2: VN stimulation after complete dissection of the lateral thyroid lobe (VN postdissection)

Intraoperatively, the device produces sound signal of motor electrophysiological activity, while the EMG signal is recorded as wave amplitude. Sound signal and recording of electromyographic amplitude (as *μ*V) represent the proper function of the RLN.

The surgical anatomy, visual identification, and integrity of the RLN were established using careful surgical exposure throughout the cervical course of the nerve. The motor activity, functional identification, and integrity of the RLN were determined by IONM.

Preoperative and postoperative laryngoscopic examinations were performed on all patients to determine VCs activity. We studied demographic features of patients, indications for secondary surgery, anatomy of the RLN and anatomical changes secondary to recurrent goiter mass and prior surgery, results of IONM and functional integrity of the RLNs, and results of surgery and postoperative outcome of patients and pathological findings.

## 3. Results

47 of our patients (96%) were female. The average age of patients with recurrent goiter was 46 years (range: 31–64 years).

49 patients with recurrent goiter were surgically treated in this study. Indications for redo thyroid surgery were multinodular goiter (MNG) in 19 patients (38.8%). In addition to both physical examination and imaging findings, results of cytology from thyroid nodules in recurrent goiter mass indicated surgery in 14 patients (28.5%). Similarly, parathyroid adenoma was a surgical indication for redo surgery in 5 patients (10.2%) in whom concurrent nodular goiter was also excised (Table 1).

Recurrent goiter masses were removed by redo thyroid surgery. The mean time interval between primary thyroid surgery and thyroid reoperations was 23.4 (range: 7–45) years (Table 2). On these patients, 12 total thyroidectomies and 26 right and 11 left hemithyroidectomies were performed. Therefore, 38 right and 23 left (in total 61) lateral lobes were explored, dissected, and completely excised. We laterally approached 41 thyroid lobes (67.2%) between the sternocleidomastoid (SCM) and sternohyoid (SH) muscles because of heavy scarring in the midline.

**Figure 1:**
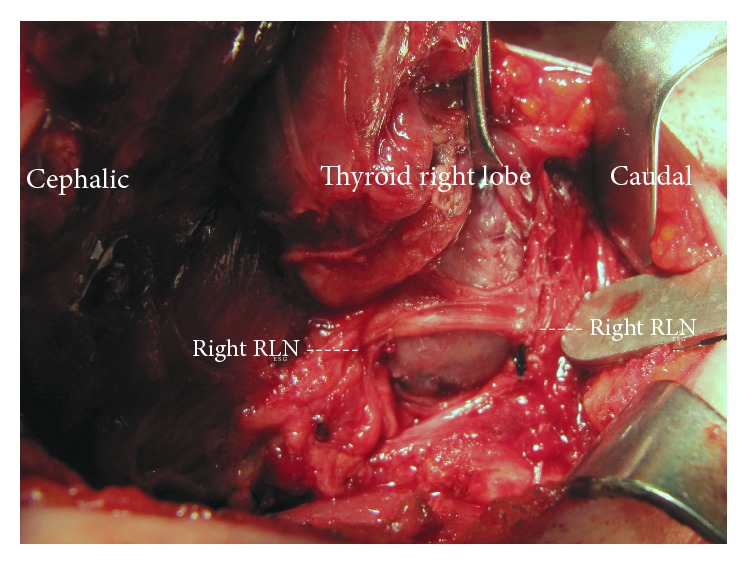
Displacement of the right recurrent laryngeal nerve (RLN) adherent to the lateral surface of recurrent goiter mass.

**Figure 2:**
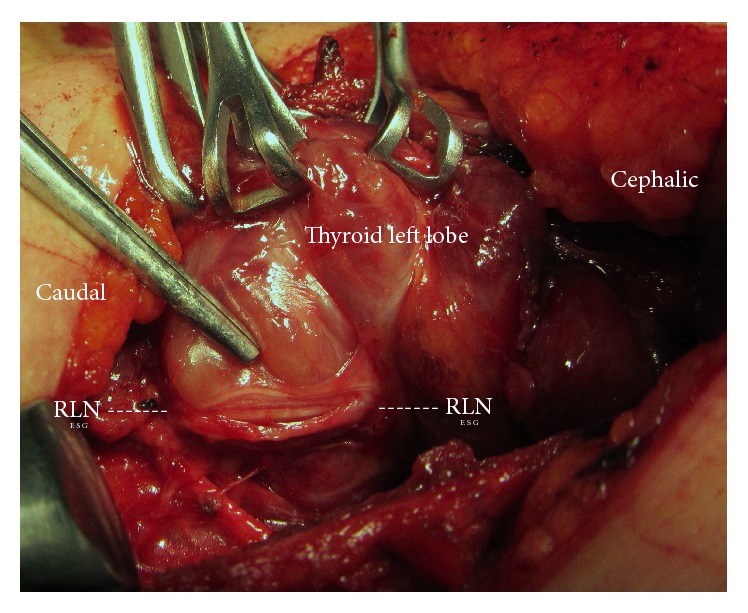
The left recurrent laryngeal nerve (RLN) adherent to the lateral surface of recurrent goiter mass.

The cervical parts of 61 RLNs (38 right and 23 left) were visually identified and totally exposed along their cervical courses. We found both lateral and superficial courses of the nerve on recurrent thyroid masses in many patients with thyroid reoperations (Table 3). 16 RLNs (26.2%) were found to be adherent to the lateral surface of the corresponding thyroid lobe (Figures 1 and 2). All RLNs were also functionally identified by IONM. The functional integrity of such nerves was confirmed by IONM before, during, and after dissection of thyroid lobes with R1 and R2 wave amplitudes (Table 3). In these series, postoperative period was uneventful. Pathology reports revealed papillary cancer in 8 (16.3%) patients (Table 1).

## 4. Discussion

Today, total thyroidectomy provides radical surgical management of diseases without leaving any thyroid tissue behind so that the risk of recurrent goiter is completely eliminated. Cappellani et al. [5] have mentioned that the best management of recurrent goiter is through its prevention by primary total thyroidectomy. Total thyroidectomy is an operation that can be performed safely, with a low incidence of permanent complications, thus avoiding future recurrences and reoperations [6]. Although many surgeons now opt for a total thyroidectomy, classically a bilateral subtotal thyroidectomy was performed to minimize operative risk and leave a small portion of functioning tissue [1]. Unfortunately, subtotal thyroidectomy carries a considerable lifelong risk of recurrence for some patients after a long interval. After subtotal resection of multinodular goiter, rates of up to 40% have been reported for recurrent goiter at long-term follow-up [7]. Conservative surgery of the thyroid is followed by recurrence in 2% to 70% of cases over a period of 8–20 years. The surgical treatment of such recurrences is affected by higher morbidity than a primary total thyroidectomy [5]. In cases of recurrent surgical pathology, scarred and disturbed anatomy due to previous primary surgery requires a complicated dissection during redo surgery which may increase the risk of surgical complications. In our present series, heavy scarring in the midline urged us to use a lateral approach to gain access to the thyroid lobes in a considerable numbers of patients.

Surgery for recurrent nodular goiter is associated with a significant risk of RLN morbidity [8]. Injury to laryngeal nerves, especially RLN and VCs palsy, are the most important causes of complications from a medicolegal perspective. The RLN is vulnerable to injury in thyroid reoperations because of the presence of scar tissue and displacement of the nerve from its normal position [9]. Richer and Wenig [1] have mentioned that technical challenges in reoperations have resulted in treating thyroid disease completely at the time of the initial operation. However, in circumstances of residual or recurrent disease, thyroid reoperations are unavoidable. In our present series of secondary surgical procedures, we determined significant changes in surgical anatomy. Complete knowledge of anatomical disturbances due to previous surgery and identification of disturbed structures during redo surgery may minimize surgical complications. Because of anatomic distortion, a lateral approach to the thyroid remnant may be used. This approach allows access along the medial border of the sternocleidomastoid muscle, enabling the surgeon to remain lateral to the strap muscles rather than accessing the thyroid bed through the anterior midline scar. In the present series, a lateral approach to the thyroid bed between the SCM and the SH muscles in two-thirds of thyroid lobes revealed the challenging and complicating effects of fibrosis, scar tissue, and wound contracture.

Our results of diagnosis leading to thyroid reoperations showed that the indications for redo surgery were similar to primary cases: symptomatic nodular goiter, thyrotoxicosis, and cytology reports. Large, symptomatic solitary or multiple nodules constituted the majority (53.1%) of our surgical indications, followed by cytology results (28.5%) from thyroid nodules that the cytology results were atypia or follicular lesions of undetermined significance, indeterminate lesions, or lesions suspicious for malignancy. Hyperthyroidism (8.2%) was another cause of recurrent pathology in the remnant thyroid. Barczyński et al. [4] have reported nodular disease (67.4%), malignant or suspicious cytology results (28.3%), and thyrotoxicosis (4.2%) as indications for redo surgery. Indications for reoperation after thyroidectomy fall into three broad categories: malignancy, multinodular goiter, and thyrotoxicosis. Also, other inflammatory conditions of the thyroid gland unresponsive to medical management are additional indications for reoperation [1]. In our series, besides thyroidal lesions, hyperparathyroidism (parathyroid adenoma) leading to surgery is another indication for reoperations which required secondary intervention in the thyroid bed. Our final pathology examinations after secondary surgery have reported papillary cancer in a considerable number of patients (8/49, 16.3%). Fortunately, 7 cases were papillary microcancer of the thyroid. These results showed that remnant thyroid after subtotal resection might cause malignant progression in some patients after a long period of time. The remnant tissue harboring the malignant lesion should be excised by redo surgery.

In our series, the mean time interval of 23.4 years between primary and redo surgery revealed the slow growth rate of thyroid pathologies as evidenced by the long period of time required for the development of a symptomatic recurrent lesion in the remnant tissue. The mean interval between the initial and the reoperative procedure has been reported to be less than 20 years (14.9, 17.5, and 18.7 years in some previous series [2, 5, 10]). The mean intervals from the first operation to recurrence were more than 20 years (24 and 27 years in other series [11, 12]). Recurrence in remnant thyroid has a slow progression rate as confirmed by 61.2% of our patients who had redo surgery after more than 20 years from primary surgery. Therefore, patients with less than total excision in initial surgery should be followed up lifelong for recurrent goiter.

The main result in the present series is that anatomical changes occur in the cervical course of the RLN. In normal cervical anatomy, it is located medially, posterior to the thyroidal lobe but lateral, and superficial course of the nerve is uncommon. Sometimes the presence of an enlarged Zuckerkandl's tubercle (ZT) may affect the course of the RLN as an anatomical variation in the virgin neck. A larger ZT is present in 51% of thyroid lobes, and RLN was laying on the anterior, lateral surface of the ZT in 6% of instances [13]. The incidence of a superficial, lateral course of the RLN has been reported as 6%-7% in primary thyroidectomy cases [14, 15]. However, our observations during redo surgery show that wound contracture, fibrosis, and scar tissue significantly affect structural anatomy in the thyroid bed including RLN anatomy. In addition, based on our results, we can comment that the gradual growth of the recurrent goiter mass and fibrotic process after previous surgery may disturb the anatomy of the RLN. Thyroid reoperations increase the risk of injury to the RLN because of the presence of scar tissue and displacement of the nerve from its normal position [9]. Our results revealed significant anatomical disturbances in the cervical course of the RLN that more than one-quarter of identified nerves showed lateral displacement. We found the RLN in a lateral location according to remnant tissue when compared with normal anatomy. Identification and preservation of the RLN are the most critical challenges during thyroid reoperation, since both can be difficult as a result of the change in the location of the RLN [1]. In thyroid reoperations, the RLN may be displaced in any direction. The nerve is dislocated from its typical course due to the remnant thyroid tissue growing close to it. RLNs adherent to the lateral capsule of a goiter are most frequently seen in the recurrent mass many years after the first partially removed goiter [16]. In our series, 26.2% of exposed RLNs were identified at both lateral and superficial locations in the thyroid bed according to remnant tissue. Based on our results, we can comment that in reoperations the RLN could be displaced laterally and located adherent to the lateral capsule of the remnant thyroid. In this position, the nerve is stretched by the enlarged remnant and may be mistaken for fibrotic bands, thin ligaments, and blood vessels, thereby increasing the risk of inadvertent transection if not identified properly. We used IONM in all cases of secondary surgery and found it to be a good adjunct to the usual anatomical identification and surgical dissection for functional confirmation of its presence. Functional identification of the nerve at the beginning of surgery (R1) confirmed its visual identification, which is the gold standard in thyroid surgery. At the end of surgery, the anatomical integrity of the nerve was supported with verification of functional integrity (R2 and V2) by IONM. In the situation of disturbed anatomy, new ancillary methods such as IONM help to identify the nerve and justify its functional status during redo surgery. The nerve adherent to the lateral capsule was dissected completely from the recurrent goiter mass to provide total resection. After this challenging dissection from the lateral surface of an enlarged remnant, confirmation of the functional integrity with nerve monitoring increases safety of thyroidectomy. The IONM provides intraoperative information about RLN function and helps to predict its postoperative functional status. Wojtczak and Barczyński [16] have reported that final confirmation of RLN functionality is crucial in cases where the RLN runs over a recurrent goiter because the nerve is particularly prone to overstretching with the potential loss of function after dissection. We used a lateral approach to the thyroid bed between the SCM and the SH muscles in the majority of our cases. Proper, careful surgical technique and both anatomical and electrophysiological identification of the RLN in all our patients provided the performance of redo surgery without neural complications. Sun et al. [17] have reported in a meta-analysis that IONM is associated with a reduction in overall and permanent RLN palsy in thyroid reoperations. A potential nonanatomical RLN course shows that the lateral approach seems to be the most useful technique in repeat thyroid operations. In secondary operations, the functional integrity of the nerve should be confirmed with IONM. The prevalence of permanent RLN injury tended to be lower in thyroid reoperations with IONM [4, 16]. On the other hand, extensive experience in thyroid surgery should still be considered as the most important factor for approaching redo thyroid surgery. The routine use of IONM does not seem to reduce the incidence of RLN injury during redo thyroid surgery [2, 18].

In conclusion, a symptomatic recurrent goiter may occur after a long period of time following primary surgery of less than a total thyroidectomy. The development of surgical pathology in the remnant tissue can then lead to goiter recurrence requiring redo surgery. Scar tissue, fibrosis, and wound contracture due to previous surgery complicate surgical dissection during reoperations. In this situation, a lateral approach to the thyroid bed avoiding the midline scar is advisable for operative safety. Surgeons should be aware of considerable disturbances in RLN anatomy. Lateral displacement of the RLN course is common. It is found to be adherent to the lateral surface of the remnant tissue. Visual identification of the RLN is also the gold standard in redo surgery in which IONM seems like a good adjunct to surgical dissection, especially for uncommon situations like distorted anatomy after previous surgery. Redo thyroid surgery can be performed safely if it is based on

- complete anatomical knowledge including primary variations and secondary distortion due to initial operation,
- surgical experience and skillful technique,
- awareness of anatomical disturbances after primary surgery,
- support of ancillary electrophysiological technology.

## Figures and Tables

**Table 1:** Preoperative diagnosis and indications for thyroid surgery in patients with recurrent goiter, surgical procedures, and final malignant diagnosis.

Indications for redo surgery	%	Patients	Surgery^¶^			Pathology^¶¶^
			TT^¶^	RH^¶^	LH^¶^	
MNG^*∗*^	38.8	19	7	9	3	P Ca (1) PTM (2)
Solitary nodule	14.3	7	--	5	2	
Hyperthyroidism (4)	8.2					
Toxic MNG		2	1	1	--	
Toxic adenoma		2	--	2	--	
MNG associated with hyperparathyroidism	10.2	5	1	3	1	PTM (1)
Cytology	28.5					
Follicular lesion		6	2	2	2	PTM (2)
Hurthle cell lesion		2	--	2	--	PTM (1)
AUS/FLUS^*∗∗*^		6	1	2	3	PTM (1)

Total		49	12	26	11	P Ca (8)

^*∗*^MNG: multinodular goiter; ^*∗∗*^AUS/FLUS: atypia of undetermined significance/follicular lesion of undetermined significance; ^¶^TT: total thyroidectomy; RH: right hemithyroidectomy; LH: left hemithyroidectomy; ^¶¶^P Ca: papillary cancer; PTM: papillary thyroid microcancer.

**Table 2:** Time interval between primary and redo surgery.

Time interval (years)	Patients	%
0–10	2	4.1
11–20	17	34.7
21–30	19	38.8
31–40	9	18.3
40+	2	4.1
Mean 23.4	49	100.0

**Table 3:** Cervical course of recurrent laryngeal nerves and mean wave amplitude levels.

Redo surgery	Patients	RLNs	RLNs (number)	RLNs adherent to lateral surface of thyroid remnant	IONM	Mean wave amplitude
TT^*∗*^	12	Right RLN Left RLN	38 23	**11 (28.9%)** **5 (21.7%)**	R1 R2	882.7 (263–1989) *μ*V 1064.3 (318–2064) *μ*V
RH^*∗*^	26					
LH^*∗*^	11					

Total	49		61	**16 (26.2%)**		

^*∗*^TT: total thyroidectomy; RH: right hemithyroidectomy; LH: left hemithyroidectomy.
